# OncoBase: a platform for decoding regulatory somatic mutations in human cancers

**DOI:** 10.1093/nar/gky1139

**Published:** 2018-11-16

**Authors:** Xianfeng Li, Leisheng Shi, Yan Wang, Jianing Zhong, Xiaolu Zhao, Huajing Teng, Xiaohui Shi, Haonan Yang, Shasha Ruan, MingKun Li, Zhong Sheng Sun, Qimin Zhan, Fengbiao Mao

**Affiliations:** 1 Key laboratory of Carcinogenesis and Translational Research (Ministry of Education/Beijing), Laboratory of Molecular Oncology, Peking University Cancer Hospital & Institute, Beijing 100142, China; 2 Beijing Institutes of Life Science, Chinese Academy of Sciences, Beijing 100101, China; 3 Key Laboratory of Genomic and Precision Medicine, Beijing Institute of Genomics, Chinese Academy of Sciences, Beijing 100101, China; 4 Key Laboratory of Prevention and Treatment of Cardiovascular and Cerebrovascular Diseases of Ministry of Education, Gannan Medical University, Ganzhou 341000,China; 5 Department of Pathology, University of Michigan, Ann Arbor, MI 48109, USA; 6 Sino-Danish college, University of Chinese Academy of Sciences, Beijing 100049, China; 7 Department of Clinical Oncology, Renmin Hospital of Wuhan University, Wuhan, Hubei 430072, China

## Abstract

Whole-exome and whole-genome sequencing have revealed millions of somatic mutations associated with different human cancers, and the vast majority of them are located outside of coding sequences, making it challenging to directly interpret their functional effects. With the rapid advances in high-throughput sequencing technologies, genome-scale long-range chromatin interactions were detected, and distal target genes of regulatory elements were determined using three-dimensional (3D) chromatin looping. Herein, we present OncoBase (http://www.oncobase.biols.ac.cn/), an integrated database for annotating 81 385 242 somatic mutations in 68 cancer types from more than 120 cancer projects by exploring their roles in distal interactions between target genes and regulatory elements. OncoBase integrates local chromatin signatures, 3D chromatin interactions in different cell types and reconstruction of enhancer-target networks using state-of-the-art algorithms. It employs informative visualization tools to display the integrated local and 3D chromatin signatures and effects of somatic mutations on regulatory elements. Enhancer-promoter interactions estimated from chromatin interactions are integrated into a network diffusion system that quantitatively prioritizes somatic mutations and target genes from a large pool. Thus, OncoBase is a useful resource for the functional annotation of regulatory noncoding regions and systematically benchmarking the regulatory effects of embedded noncoding somatic mutations in human carcinogenesis.

## INTRODUCTION

Noncoding variants are capable of causing common diseases and account for the vast majority of heritability (1). To date, a majority of studies have focused exclusively on the effects of missense variants in coding regions (2–4) that comprise <2% of the human genome (5). Mapping variants to the whole genome indicate that disease-associated single nucleotide polymorphisms (SNPs) are strongly enriched in regulatory elements, especially those activated in relevant cell types (6). Moreover, numerous studies have shown that associated variants for a particular trait/disease are significantly enriched in certain regulatory regions of relevant tissues/cell types (7). Importantly, the noncoding regions possess many functional elements based on one dimensional (1D) epigenomic features and three-dimensional (3D) spatial long-range interactions that could help to build accurate enhancer-promoter regulatory pairs; therefore, integrating noncoding variants with 1D coordinated epigenetic profiles and 3D long-range interactions in specific tissue/cell types will provide a promising direction to fine-map causal regulatory variants and understand underlying regulatory mechanisms in human diseases.

Recent discoveries, including the identification of recurrent somatic mutations in the TERT promoter in multiple cancer types (8–11), have supported the idea that somatic mutations in noncoding regions also play vital roles in tumor development (12,13). More than 98% of somatic mutations in most cancers are located in non-coding regions, and some have been identified as putative driver mutations (14). Several databases and computational tools have been developed for annotating noncoding SNPs based on their local genomic 1D features (15–19) and/or their 3D chromatin interactions (20–24), but few tools for annotating noncoding somatic mutations have been designed specifically for human cancers. Moreover, most regulatory elements are widely dispersed across the genome (21), and regulatory somatic mutations are highly outnumbered by neutral passenger mutations due to intratumoral heterogeneity (25,26). Therefore, it is challenging to interpret the effects of noncoding somatic mutations in regulating their target genes in human cancers.

Fortunately, ENCODE (27), Roadmap Epigenomics (28) projects and studies on individual groups (29–31) have revealed the landscape of 1D regulatory elements across the human genome. The rapid development of chromosome conformation capture (3C)-based technologies, such as ChIA-PET (32,33), 5C (34) and Hi-C (35–37), has provided increased datasets on the 3D architecture of the human genome. Studies based on these technologies have uncovered models on how regulatory elements regulate the expression of distal target genes (36,38,39). Regulatory elements, such as enhancers, insulators and protein-binding sites, are anchored to the promoter regions of genes via chromatin looping to orchestrate gene transcription. Chromatin loops identified by Hi-C frequently link enhancers to promoters and are conserved across human cell lines (36) and tissues (38). In addition, enhancer-like elements frequently contact transcriptionally active genes, while potential long-range silencers interact with transcriptionally inactive genes. Furthermore, the interacting loci are enriched for disease-associated variants, suggesting that distal somatic mutations may disrupt the regulation of relevant genes (39). Recent studies have made strong cases for using 3D genome information to interpret noncoding, disease-associated variants (39–43). A system-level understanding of how cancer mutations affect signaling networks is pivotal for interpreting the complex genotype-to-phenotype relationship in terms of tumor behavior and patient outcomes (44). This sophisticated, functional understanding of somatic mutations is key for distinguishing driver mutations from non-pathogenic passengers (26,45). Therefore, it is essential to link noncoding regulatory somatic mutations to target genes by integrating 3D chromatin interactions and 1D chromatin signatures.

**Figure 1.**
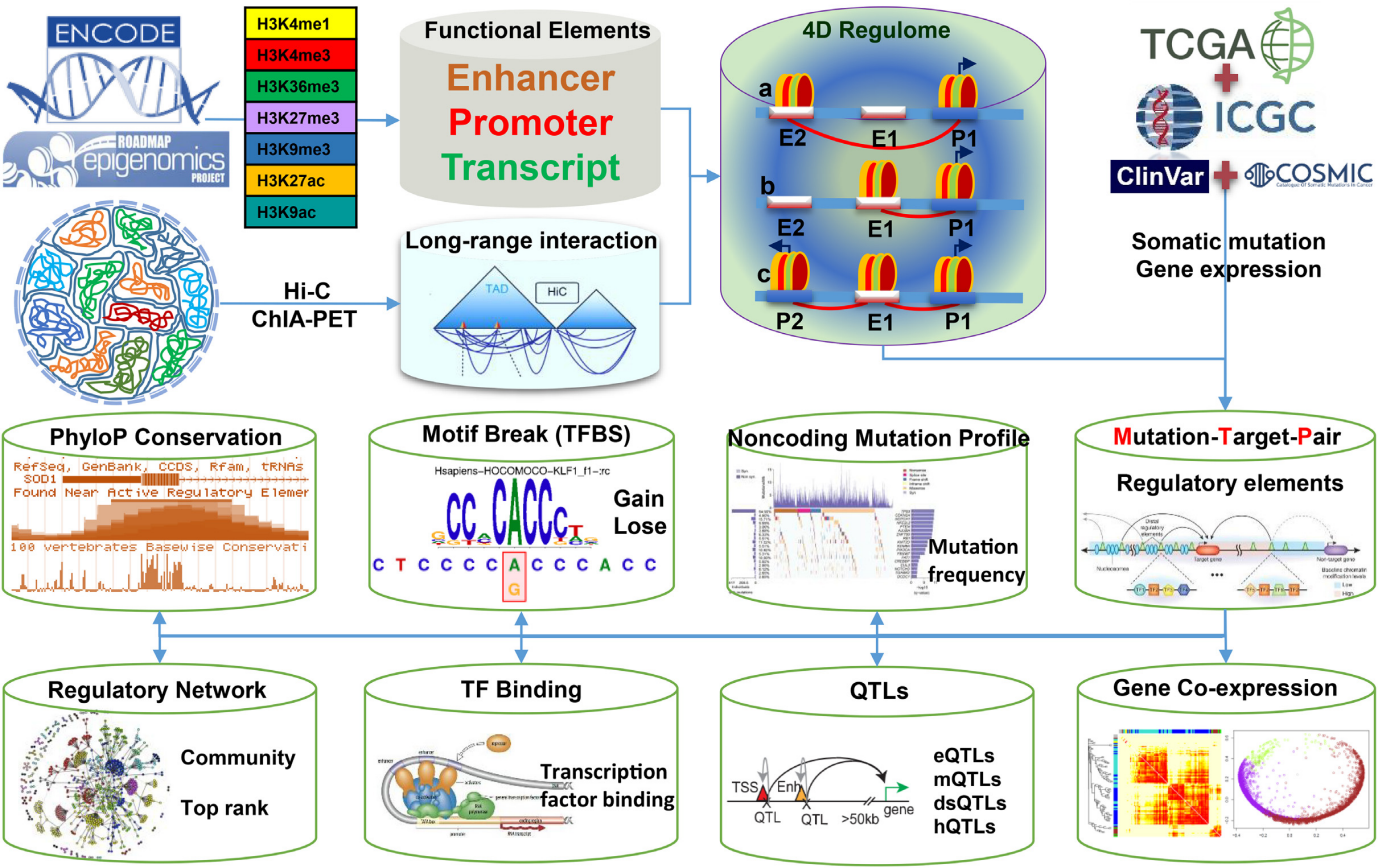
Workflow of OncoBase construction.

A large number of tumor somatic mutations have been identified by TCGA (46), ICGC (47), COSMIC (48) and ClinVar (49,50) but the potential functions of most of these noncoding somatic mutations remain unknown. In this study, we built the platform **OncoBase** to decipher tum**O**r **N**on**C**oding s**O**matic mutations by **B**ase-p**A**ir re**S**olution **E**stimation (Figure 1). OncoBase provides comprehensive annotations and predictions of regulatory somatic mutations by employing state-of-the-art methods for target predictions, gene or mutation prioritizations and functional predictions. OncoBase integrates genotype data, phenotype data, 3D chromatin interactions, and important genomic features, including chromatin states, histone modifications, gain/loss of TFBS motifs, and multiple concepts of QTLs (eQTL, dsQTLs, hQTLs and mQTLs), across a broad range of cell types. OncoBase provides a series of informative tables, publishable figures and a network diffusion scoring system to help researchers discover the regulatory roles of noncoding somatic mutations in human cancers based on their 1D and 3D genomic features.

## MATERIALS AND METHODS

### Somatic mutations and tumor types

To curate as many somatic mutations as possible, we collected somatic mutations from four databases (Table 1), including 1 823 191 somatic mutations in 36 cancer types from TCGA (46), 77 460 941 somatic mutations in 57 cancer types from ICGC (47), 20 909 477 somatic mutations from COSMIC (48), and 345 756 clinical variants from ClinVar (49,50). In total, we collected 81 385 242 somatic mutations in more than 120 types of cancer projects. These somatic variants including noncoding somatic mutations and coding somatic mutations were annotated by ANNOVAR (51).

**Table 1.** Summary of data sources of OncoBase

Type	Source	Cell-types/tissues	Number of regions/sites	Record of mutation	#Unique mutation
**Somatic mutation**	ICGC	57	-	77 460 941	81 385 242
	TCGA	36	-	1 823 191	
	COSMIC	-	-	20 909 477	
**Mutation**	ClinVar	-	-	345 756	345 756
**Regulatory elements**	ENCODE	16	3 824 829	21 640 293	57 344 038
	RoadMap	111			
	Cistrome	352	133 638 513	44 139 519	
	GTRD	1 355	38 291 345	28 171 788	
**Mutation_Target_Pairs**	EpiTensor	127	394 060	7 371 607	49 110 107
	JEME	127	288 882	6 793 562	
	GeneHancer	-	233 757	9 065 807	
	4DGenome	53	1 151 004	45 199 484	
**Enhancer/promoter**	EnhancerAtlas	71	577 992	22 452 362	30 145 302
	dbSUPER	99	65 213	11 435 320	
	SEA	15	2 283	548 744	
	UCNEbase	-	4 315	35 389	
	HoneyBadger (enhancer)	127	1 598 323	7 023 417	
	HoneyBadger (promoter)	127	56 893	1 063 507	
**QTL**	GTEx_eQTL	48	341 316	86 548	3 190 193
	PancanQTL	33	1 412 029	647 927	
	Other eQTL	9	2 612 515	536 714	
	mQTL	5	14 217 993	3 135 075	
	dsQTL	1	214 522	59 782	
	hQTLs(distal)	1	9 972	244	
	hQTLs(local)	1	37 287	13 070	
**Motif**	motifbreak	-	-	74 141 414	74 141 414
**Expression**	TCGA	36	-	-	-
	GTEx	48	-	-	-
**Conservation**	UCSC	-	-	-	-

### One-dimensional chromatin features

A variety of local chromatin signatures were used to annotate the regulatory functions of somatic mutations, including predicted chromatin states, histone modifications, DNase I hypersensitivity sites (DHSs) and transcription factor binding sites (TFBSs). Chromatin states were predicted using the core 25-state ChromHMM model (52,53) trained on the imputed data for 12 marks, H3K4me1, H3K4me2, H3K4me3, H3K9ac, H3K27ac, H4K20me1, H3K79me2, H3K36me3, H3K9me3, H3K27me3, H2A.Z and DNase I hypersensitive sites (DHSs), across all 127 reference epigenomes. Regulatory somatic mutations were assigned to corresponding enhancers or promoters with chromatin states by intersecting intervals by coordinate. The binding sites of 153 transcription factors in 91 human cell lines and DHSs in 125 cell lines were obtained from ENCODE and intersected with somatic mutations for annotation. In addition, 1034 epigenetic profiles of 29 main human tissues or cells were implemented to allow for a well-organized visualization by using the JBrowse Genome Browser.

### Prediction of the effects of somatic mutations on transcription factor binding

First, 2 817 position weight matrices (PWMs) of transcription factors from the HOCOMOCO (54), FactorBook (55), Homer (56) and ENCODE motif (57) were collected by motifbreakR (58) were used for further prediction of the effects of somatic mutations. In contrast to 3DSNP (21), we employed motifbreakR (58) to measure the effects of somatic mutations on TF binding motifs by using a highly efficient information content-based algorithm to discriminate between truly disruptive *versus* neutral variants. In contrast to the TFM-Scan software employed by 3DSNP (21), motifbreakR scores and reports the reference and alternate alleles of the sequence and the effect (strong, weak or neutral) according to *P*-values for PWM match.

### Sequence conservation of regulatory somatic mutations

The conservation of somatic mutations was measured by the PhyloP scores obtained from the UCSC Genome Browser (59). The PhyloP scores were calculated from multiple alignments of 100 vertebrate genomes. The absolute values of the PhyloP scores represent -log(*P*-values) under a null hypothesis of neutral evolution, and sites predicted to be conserved are assigned positive scores, while sites predicted to be fast-evolving are assigned negative scores.

### Reconstruction of high-resolution 3D chromatin interactions

Hi-C sequencing datasets were chosen as the main sources for deciphering 3D chromatin interactions in OncoBase because Hi-C measures all pair-wise interaction frequencies across the entire genome, and detection is not dependent on any specific transcription factor. As the linear increase in resolution requires a quadratic increase in the total number of sequencing reads as well as sequencing cost, most available Hi-C datasets have a relatively low resolution, such as 25 or 40 kb. These low-resolution Hi-C datasets can be used to define large-scale genomic patterns, such as A/B compartments or topologically associating domains (TADs), but cannot be used to identify more refined structures, such as enhancer–promoter interactions or sub-domains (60). Therefore, it is urgent to reconstruct chromatin interactions at the gene level with less than a 1 kb resolution. Here, we employed a novel algorithm named EpiTensor (61) to reconstruct chromatin interactions to investigate the regulatory roles of somatic mutations located in interacting loci. EpiTensor can capture spatial associations between distal loci at a 200 bp resolution by using tensor decomposition analysis of TADs and multi-dimensional epigenomes. To obtain higher resolution chromatin interactions of the human genome in different cell lines or tissues, we collected 80 TADs and 127 epigenomes of different cell lines or tissues from the 3DIV database (24) and RoadMap epigenomics project (28), respectively. The spatial and epigenomic datasets were then used to reconstruct chromatin interactions by EpiTensor. Finally, high-resolution interactions were constructed and classified into three types of TSS to enhancer, TSS to TSS, and enhancer to enhancer in 127 cell lines or tissues. Furthermore, high-resolution interactions were marked by 25 chromatin states predicted by ChromHMM to determine whether they are active, inactive or poised. In addition, we also collected 1 981 153 chromatin interacting pairs from the 4DGeneome (62) to expand the annotation of chromatin interactions from 53 tissues or cells by 3C, 4C, 5C, ChIA-PET and IM-PET.

### Tissue/cell type-specific enhancers/promoters and super-enhancers

Thanks to the rapid development of high-throughput sequencing technology, genome annotation consortia—e.g. ENCODE (27) and NIH Epigenome Roadmap (28)—have generated massive amounts of different types of sequencing data, making it possible to identify enhancers on a genome-wide scale. The current release of OncoBase enables the availability of a total of 30 145 302 total putative enhancers/promoters related to somatic mutations collected from 5major databases of enhancers/promoters or super-enhancers: 577 992 enhancers in 71 tissues or cell types from EnhancerAtlas (63), 65 213 super-enhancers in 99 tissues or cell types from dbSUPER (64), 2283 super-enhancers in 15tissues or cell types from SEA (65), 4 315 ultra-conserved non-coding elements (UCNEs) that typically function as enhancers in various developmental contexts (66) from UCNEbase (67), and 1 598 323 enhancers and 56 893 promoters from HoneyBadger of Reg2Map project.

### Targets of tissue/cell type-specific enhancers and super-enhancers

Although the databases mentioned here have been set up for enhancers in the human genome, they provide only limited, basic information about enhancers, such as their coordinates, cell or tissue types, and nearby genes; therefore, we employed EpiTensor to obtain 25 222 085 high-resolution (∼200 bp) chromatin interactions, including 2 847 794, 5 691 699, and 16 682 592 interactions for promoter to promoter, enhancer to promoter and enhancer to enhancer, respectively. Moreover, we curated predictions of target genes by other two algorithms: 9 879 737 enhancer-target networks in 935 samples by JEME (68) and 284 834 links of enhancers to genes by GeneHancer (69). In total, we deposited 35 386 656 enhancer-target pairs, including 19 472 521 enhancer-promoter pairs, from more than 1000 human samples.

### Interactive circular visualization of various biological data

The high-resolution chromatin interactions, clusters of transcription factor binding, somatic mutations, enhancers or super-enhancers and their predicted targets were illustrated in a circular ideogram layout by BioCircos (70), which is a useful tool implemented to circular visualization of various biological data, such as genomic features, genetic variations, gene expression and biomolecular interactions.

### Expression of quantitative trait loci

The effects of genetic variants on gene regulation could be interpreted by correlations between genotype and tissue-specific gene expression levels. Expression quantitative trait loci (eQTLs) are genomic loci that regulate gene expression levels and play a crucial role in deciphering gene regulation and spatio-temporal specificity (71). We collected a total of 341 316 significant SNP-gene pairs (FDR ≤ 0.05) in 48 human tissues from the GTEx project version 7 (72). Nominal eQTL *P*-values and the effect sizes were obtained for each SNP-gene pair to measure the significance of eQTLs. Nominal eQTL *P*-values were generated using a two-tailed t test to test the alternative hypothesis that the beta deviates from the null hypothesis of *β* = 0. The effect size of the eQTLs is defined as the slope (‘*β*’) of the linear regression and is computed as the effect of the alternative allele (ALT) relative to the reference allele (REF) in the human genome. Most importantly, we collected 1 412 029 significant cis-eQTLs- and trans-eQTLs-gene pairs in 33 cancer types from PancanQTL database (73). In addition, Oncobase also included eQTLs from experimentally supported eQTL databases (74,75) and the eQTL browser (http://eqtl.uchicagoedu/cgi-bin/gbrowse/eqtl/) (76) to provide association labels for somatic mutations. Tissue and developmental-stage information were labeled according to the cell type from which eQTL was identified. The statistical test to measure significance is similar to that used for GTEx eQTLs.

### Methylation, DNase I sensitivity and histone markers quantitative trait loci

The effects of genetic variants on DNA methylation, DNase I sensitivity and histone modifications could also aid in deciphering the function of regulatory somatic mutations in epigenetic regulation and molecular processes. Genomic loci that affect DNA methylation, DNase I sensitivity and histone modifications are called mQTLs, dsQTLs and hQTLs, respectively. DNA methylation contains significant heritable components that are highly stable across the lifespan and may have a causal role in complex traits (77). We used 14 217 993 mQTL-CpG pairs of human blood at five different life stage from mQTLdb (FDR<0.05) (77) to annotate regulatory somatic mutations. As DNase I sensitivity QTLs are a major determinant of human expression variation, we collected 214 522 dsQTL-peak pairs (FDR<0.1) in lymphoblastoid cell lines (LCLs) to annotate regulatory somatic mutations (78). In addition, we annotated the regulatory somatic mutations by using 47 259 hQTL-peak pairs (FDR<0.1) for three histone markers in LCLs, as hQTLs enable the identification of putative target genes of disease-associated variants from genome-wide association studies (79). The statistical test to measure significance is similar to that used for GTEx eQTLs.

### Gene expression in human cancers and normal tissues

The gene expression profiles in human cancers were obtained from the TCGA data portal (https://gdc-portal.nci.nih.gov/) (46), which contains 20 531 genes for each sample. In total, we collected expression data from 13 250 tumor samples in 36 cancer types from TCGA. For each cancer type, weighted gene co-expression network analysis was performed as described below. As the normal control, we collected gene expression data for 53 normal human tissues from the genotype-tissue expression project (GTEx) (72). Both the gene expression of human cancers and normal tissues were displayed in bar plot figures from the searching results.

### Weighted gene co-expression network analysis of 36 tumor types

Co-expression analysis is a powerful method for the identification of genes involved in the same molecular processes and regulating relationships. Weighted gene co-expression network analysis (WGCNA) (80) was performed to understand the co-expression relationships between genes for 36 tumor types by using the pipeline we previously employed (81). Genes with a null expression <80% in all samples in each tumor type were selected for WGCNA analysis. A step-by-step network construction workflow was employed with a soft-thresholding power value of 10 for each tumor type. A kME >0.1 was assigned to an eigengene module for co-expression networks in each tumor type. The co-expression networks were present as an interactive networks and ranked by Google PageRank algorithm. The size of the circle of each gene is positively correlated with the PageRank score.

### Network diffusion system to prioritize mutations and target genes

Google PageRank, a network-based diffusion algorithm, has emerged as the leading method to rank web content, ecological species and biology scientists (80). PageRank computes the ranking of nodes in graph G based on the structures of incoming connections. It was originally designed as an algorithm to rank web pages. But, here PageRank expresses each gene or mutation as a single item node, and an item containing one or more connections pointing to another item B indicates that item A approves the importance of item B and casts a vote for item B. This relationship can be abstracted as a directed edge in a graph structure. From the point of view of energy passing, each node will distribute its own weight to the nodes to which it points. After several rounds of iterations, diffusion system will complete its convergence and obtain each respective PageRank score. In mathematical terms, the general form of PageRank is expressed as follows:

\begin{equation*}{\rm{PR\ }}\left( {{u}} \right) = \left( {1 - d} \right)\ + d \times \mathop \sum \limits_{v \in B\left( u \right)} {\rm PR}\left( v \right)\end{equation*}

PR(*u*) represents the PageRank score of node u and B(u) represents the set of nodes that point to u. The parameter *d* is used to solve the situation in which no node points to *u*. If it is not set, then the converging value will be 0 at the end. Here, we set *d* = 0.85 for its best practice (82).

Here, the regulatory networks were considered as a graph structure by PageRank. For example, if there are 10 connections in item *A*, 9-point to item *B*, and 1 points to item *C*, then *A* should assign more weight to *B* than *A*. Therefore, a slightly improved version of the PageRank form is as follows:

\begin{equation*}{\rm{PR\ }}\left( u \right) = \left( {1 - d} \right)\ + d \times \mathop \sum \limits_{v \in B\left( u \right)} {\rm PR}\left( v \right) \times {{\rm weight}_{v \to u}}\end{equation*}

Weight_*v*→*u*_ is used to measure the weight of the edge. For the edge derived from chromatin interaction determined by EpiTensor, we assigned the weight value as 1 while we used the enhancer–target scores ([0, 1]) as the weight value from the interaction connections predicted by JEME. Finally, we provided an interactive view of periodization of genes related to certain somatic mutation in two layers’ network ranked by score of PageRank in section ‘Regulatory_Network’. PageRank ranking were applied to all genes regulated by certain somatic mutation and all of other somatic mutations regulating these genes. The network view only shows the first layer networks and the somatic mutations or genes related to second layer networks are showed in a table.

## DATABASE FEATURES AND APPLICATIONS

### Architecture and statistics

**Figure 2.**
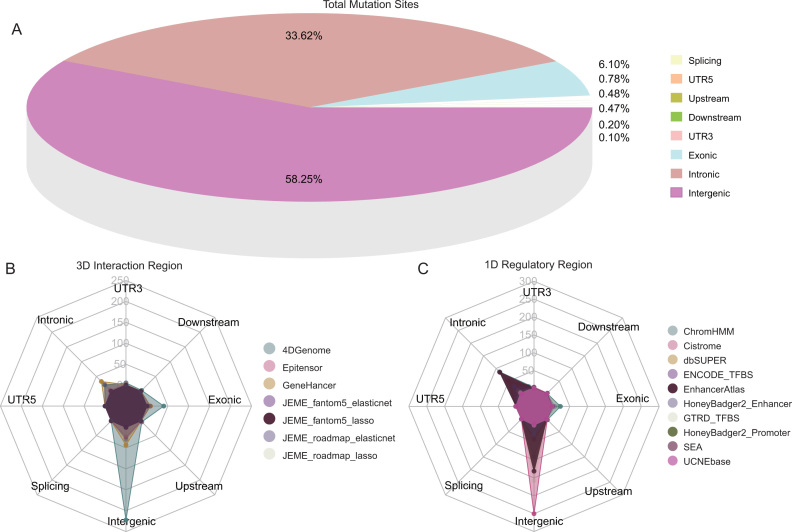
Distribution of mutations in OncoBase. (**A**). Total mutations located in eight elements annotated by software ANNOVAR. (**B, C**). Distribution of mutations located in 3D interaction regions and 1D regulatory regions.

The user-friendly web interface OncoBase (http://www.oncobase.biols.ac.cn/ or http://159.226.67.237/sun/oncobase) was developed by combining jQuery with the PHP-based web framework CodeIgniter, supported by versatile browsing and searching functionalities similar to our previous databases and webservers (71,81,83–87). Annotation information was stored in either the MySQL database or flat files. Academic users can access genetic data or extended analysis results freely via the web interface with no requirement permissions. OncoBase stored 81 385 242 somatic mutations and 345 756 clinical mutations collected from database ICGC or TCGA or COSMIC, and ClinVar, respectively. More than 90% mutations were located in intergenic or intronic regions (Figure 2A), and the majority of mutations were located in intergenic and intronic regions of both three dimension (3D) spatial long-range interactions regions and one dimension (1D) epigenomic regulatory regions (Figure 2B, C).

### Website interface

**Figure 3.**
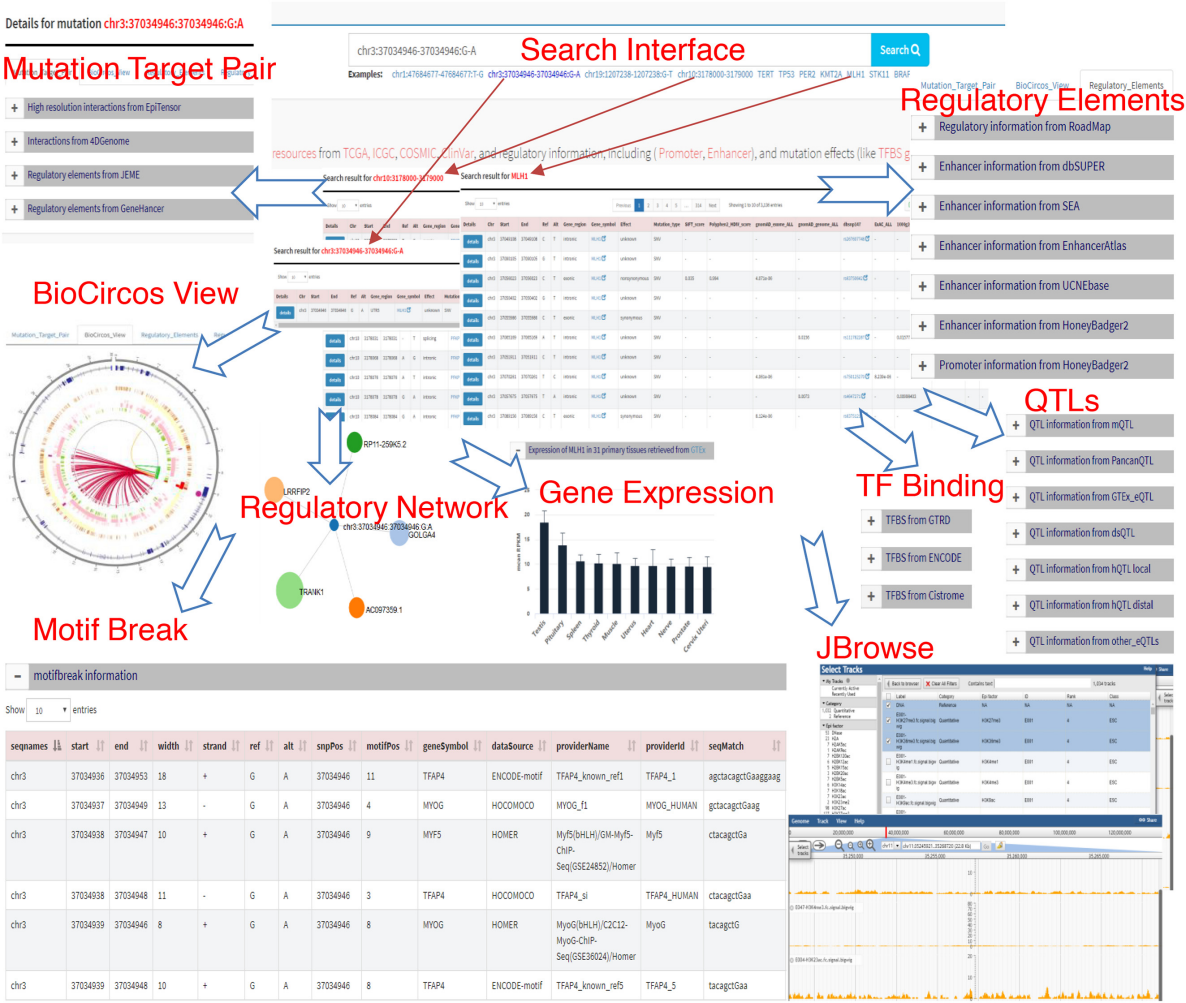
Web interface of OncoBase.

The data retrieved in OncoBase can be searched in three formats: ‘gene symbols’, ‘somatic mutations’, ‘dbSNP ID’ and ‘regulatory regions’ (Figure 3). A ‘gene symbols’ search is very useful in terms of searching for gene expression and epigenetic regulation of genes of interest in human cancers and candidate regulatory regions or somatic mutations that are based on genes. ‘Somatic mutation’ retrieval is appropriate for analyzing the results of genetic studies for human cancers and is especially useful for the results of high-throughput studies. ‘Somatic mutation’ retrieval gives support for further functional studies to identify regulatory somatic mutations and sheds light on the underlying molecular mechanisms of human carcinogenesis. In addition, OncoBase allows ‘regulatory region’ retrieval, which could elucidate the potential roles of regulatory regions by providing genomic regions to search somatic mutations that were deposited in the curated database. Furthermore, the JBrowse Genome Browser (http://jbrowse.org) was applied to establish a well-organized ‘JBrowse’ page for visualizing genome-wide signals of epigenetic data sets from 127 Roadmap epigenomes, including signals of fold change compared with input for H2A ChIP-seq, DNase-seq and 30 kinds of histone modifications measured by ChIP-seq. Users can select and browse sequencing signals from any epigenetic type and any cell or tissue across a specific genomic region or mutation of interest. Genetic mutations located in the regulatory region may provide a clue that this mutation may affect the local epigenetic status and result in dysregulation of gene expression.

### Case study

**Figure 4.**
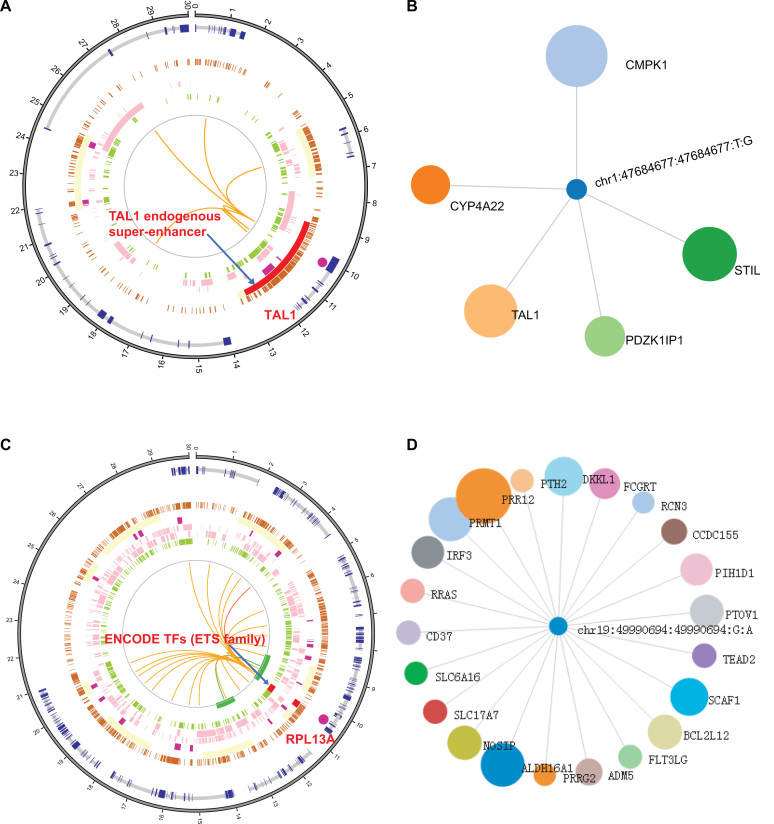
Two case studies of mutation in OncoBase. (**A**). Circos plot of 3D and 1D information related to rs977747 and chr19:49990694:49990694:G:A. (**B**). Regulatory network of mutation and its target genes produced by Google PageRank method for rs977747. (**C**) Recurrent mutation in promoter can affect binding of ETS family protein. (**D**) Regulatory network of mutation and its target genes produced by Google PageRank method for chr19:49990694:49990694:G:A.

To illustrate the usage of OncoBase, we search the database with a well-studied 3′ UTR’s somatic mutation at the chromosome 1p33 locus, rs977747 (‘chr1:47684677-47684677:T-G’), associated with T-cell acute lymphoblastic leukemia (T-ALL) (88). From the ‘BioCircos_View’ summary section, we can see that somatic mutation rs977747 is located in an endogenous super-enhancer ‘chr1:47675704-47707659’ upstream of the TAL1 oncogene (Figure 4A). In the ‘Mutation_Target_Pair’ information section, we can see five potential target genes of rs977747 based on the targets of enhancers: CMPK1, TAL1, STIL, PDZK1IP1 and CYP4A22 (Figure 4B). The prioritization of the target genes was performed by Google PageRank and showed in ‘Regulatory_Network’ section, which displays the size of genes based on the significance in the regulatory network centered on a functional mutation. According to the annotation of ‘TF_binding’ section, ESR1 binds to this position in invasive ductal breast carcinoma. More interestingly, we find that somatic mutation rs977747 is also an mQTL in the blood of different developmental stages showed in ‘Quantitative_Trait_Locus’ section. It suggests that rs977747 may regulate gene expression through affecting DNA methylation. In addition, somatic mutation rs977747 is also eQTL in blood and cerebellum with the target genes CMPK1 and STIL, respectively. Thus, this case study redisplayed a genetic mechanism responsible for the generation of oncogenic super-enhancers in malignant cells (88) and provided additional insight into the molecular functions of the noncoding somatic mutation rs977747. In addition, we also present a recurrent mutation (chr19:49990694:49990694:G-A) in the promoter of gene RPL13A and RPL13AP5 in melanoma. In according with the article reported, this mutation overlapped with ETS family protein (ELF1, ELK1, ETS1 and GABPA) from ENCODE data in our ‘TF_Binding’ part. Further, the ‘Motif_Break’ prediction results show this mutation can strong affect the binding of that family protein. In addition, ‘Mutation_Target_Pair’ show this mutation may interact with several genes such as RPL13A, ALDH16A1, RCN3 and FLT3LG in long-range distance (Figure 4C, D) (89). These information provide a hypothesis: this G/A mutation may affect TFs binding and result in differential gene expression of its target genes. Users can validate their own hypothesis by molecular and functional experiments with lots of valuable clues provided by our platform.

## DISCUSSION AND PERSPECTIVES

The expansion of functional data sets across a wide range of cell types will improve the functional predictions of noncoding variants for tissue-specific phenotypes (90). 3D chromatin interactions are crucial for deciphering the roles of regulatory elements and embedded variants (21). Recently, several data-driven methods (15,18,21–23,81,91) and sequence-based tools (92–97) have been developed to decode noncoding germline mutations from the GWASdb (65) or dbSNP (98) databases, but none of these databases were designed to investigate functional somatic mutations in noncoding elements for human cancers. And the scientific evidence on noncoding mutations being driver events in cancer remains limited. Compared with previous tools, the principal advantages of OncoBase for the annotation of regulatory somatic mutations are as follows:

1. Collected all of the somatic mutations identified by TCGA, ICGC and other somatic or clinical mutations deposited in COSMIC and ClinVar. These somatic mutations comprise noncoding variants as well as coding variants with comprehensive annotations by ANNOVAR.
2. Constructed more than 49 million enhancer–target interactions by multiple predictions from multiple resources.
3. Incorporated 127 tissue/cell type-specific epigenomes data from the ENCODE and Roadmap epigenomics project.
4. Integrated the motifs of 2817 transcriptional regulators from four public resources and predicted the effects of mutations on binding motifs.
5. Uniformly processed Hi-C sequencing data and reconstructed 25 million chromatin interactions at a high resolution across 127 tissues/cell types.
6. Provided comprehensive functional annotations and predictions of regulatory somatic mutations.
7. Equipped a highly interactive visualization function for mutation-target interactions.
8. Included multiple concepts of QTLs, including eQTLs, mQTLs, dsQTLs and hQTLs.
9. Prioritized regulatory mutations and target genes by network diffusion.
10. Established weighted gene co-expression networks for 36 tumor types.

It is still a challenge to identify noncoding driver mutations though several studies pointed out dysregulation of enhancer-promoter interaction due to somatic mutations could constitute a general mechanism of carcinogenesis (88,99–101). The widespread implementation of noncoding variant annotation methods will help predict the effects of genomic variation, elucidate the mechanisms and pathways of human cancers, and understand the full complexity of the human genome. With the discovery of clustered regularly interspaced short palindromic repeat (CRISPR) editing, the functions of noncoding variants can now be investigated more easily with these experiment-based systems (102). Currently, owe to the limited number of 1D and 3D sequencing data sets, the 1D epigenomic profiles were mainly collected from healthy cell lines/tissues. Those data sets can easily provide loss of long-range interaction resulted from somatic mutation. Whereas, more long-range interaction data sets in cancer cells are required to identify gain of long-range interaction. In addition, genome sequencing may be changing after many times of passage in the laboratory and long-range interaction may also change. Taken together, more 1D and 3D sequencing data sets are required to provide more accurate regulatory role prediction in long distance. In the future, OncoBase will be frequently updated with new Hi-C datasets and extended to other functional somatic mutations validated by experimental methods, such as CRISPR editing. We are dedicated to maintaining and improving OncoBase since it is a valuable resource for the research community. Finally, incorporation of 3D chromatin interactions will likely improve our ability to assign regulatory somatic mutations to their target genes, thus providing additional improvements to our ability to discern their functions and place them in their biological context, a necessary step for critical pharmacogenetic advancement.
